# Managing intra-articular deformity in high Tibial osteotomy: a narrative review

**DOI:** 10.1186/s40634-020-00283-1

**Published:** 2020-09-09

**Authors:** Grégoire Micicoi, Raghbir Khakha, Kristian Kley, Adrian Wilson, Simone Cerciello, Matthieu Ollivier

**Affiliations:** 1 iULS-University Institute for Locomotion and Sports, Pasteur 2 Hospital, University Côte d’Azur, Nice, France; 2 Department of Orthopedics and Traumatology, Aix Marseille University, APHM, CNRS, ISM, Sainte-Marguerite Hospital, Institute for Locomotion, Marseille, France; 3 Guys and St Thomas’ Hospitals, Great Maze Pond, London, SE1 9RT England; 4 Department of Orthopedics and Traumatology, Institute of Movement and Locomotion, St. Marguerite Hospital, 270 Boulevard Sainte Marguerite, BP 29, 13274 Marseille, France; 5 HSSH, 18-22 Queen Anne Street, London, W1G8HU England; 6 University of Winchester, Winchester, UK; 7 Marrelli Hospital, Crotone, Italy; 8 Casa di Cura Villa Betania, Rome, Italy

**Keywords:** Osteotomy, Joint line convergence angle, Soft tissue correction, Overcorrection

## Abstract

The joint line convergence angle (JLCA) has a normal range between 0° to 2°, which increases in magnitude depending on the severity and stage of osteoarthritis in the knee.

The JLCA represents the interaction of the intra-articular deformity arising from the osteoarthritis and the surrounding soft tissue laxity. Therefore, the JLCA has become a vital parameter in analysing the long leg alignment views for corrective planning before osteotomy surgery. Recent studies have considered the influence on how the preoperative JLCA is measured and its influence on achieving accurate postoperative desired correction in high tibial osteotomy surgery.

The JLCA also reflects the influence of soft tissue laxity in a lower limb malalignment and many surgeons encourage it to be taken into account to avoid non physiological correction and/or overcorrection with negatively impacted postoperative patient outcome.

This present review addressed how to obtain an accurate preoperative measurement of the JLCA, its influence on postoperative deformity analysis and how to reduce errors arising from an elevated preoperative JLCA.

We have proposed a formula to help determine the value to subtract from the planned correction in order to avoid an overcorrection when performing a corrective osteotomy.

Level of clinical evidence IV, narrative review.

## Introduction

Preoperative planning for lower limb alignment correction is essential to define tibial, femoral and intra-articular morphologies in osteotomy surgery. Various methods have been advocated to plan and perform osteotomy with optimized accuracy [3, 10, 12, 22, 32]. However, in certain cases, surgeons fail to achieve the desired level of correction due to the unpredictable influence of intra-articular deformity correction [19, 24, 26, 44, 48]. The soft tissue tension and intra-articular deformity are approximated by the joint line convergence angle (JLCA) which is rarely taken into consideration or measured intraoperatively [48].

There are two main considerations of the JLCA when performing osteotomy surgery:

Firstly, patients with preoperative JLCA higher than 3° on standing radiographs are more likely to have a discrepancy in mechanical axis between supine and standing positions [40].

Secondly, JLCA is often englobed in lower-limb deformity analysis and thus “transformed” into the bony correction during osteotomy planning.

Noyes et al. described anatomic abnormalities of the varus knees into three categories:

Primary varus refers to tibiofemoral osseous alignment and geometry, whereas double varus refers to added varus due to separation of the lateral tibiofemoral compartment by deficiency of the lateral soft tissues [34]. Finally, triple varus has similar features than double varus but includes recurvatum in extension with severe deficiency of the posterolateral ligamentous structures.

In this way the preoperative planning should not only involve the mechanical axis but also differentiate bony and intra-articular deformities due to osteoarthritis (OA) and soft tissue laxity.

As there is a lack of knowledge about the influence of soft tissue laxity and JLCA in unexpected correction errors, the purpose of this article was to review the literature and collect data about intra-articular deformity in osteotomy correction.

## Review

### How do we assess JLCA in preoperative planning?

The JLCA is defined as the angle between the tangent to the most distal part of the medial and lateral femoral condyle and the subchondral plate of the tibial plateau.

In preoperative planning, this can be measured between the lines connecting the distal femur and the proximal tibial articular surfaces on anteroposterior weightbearing long-leg radiographs [28, 36, 42].

**Fig. 1:**
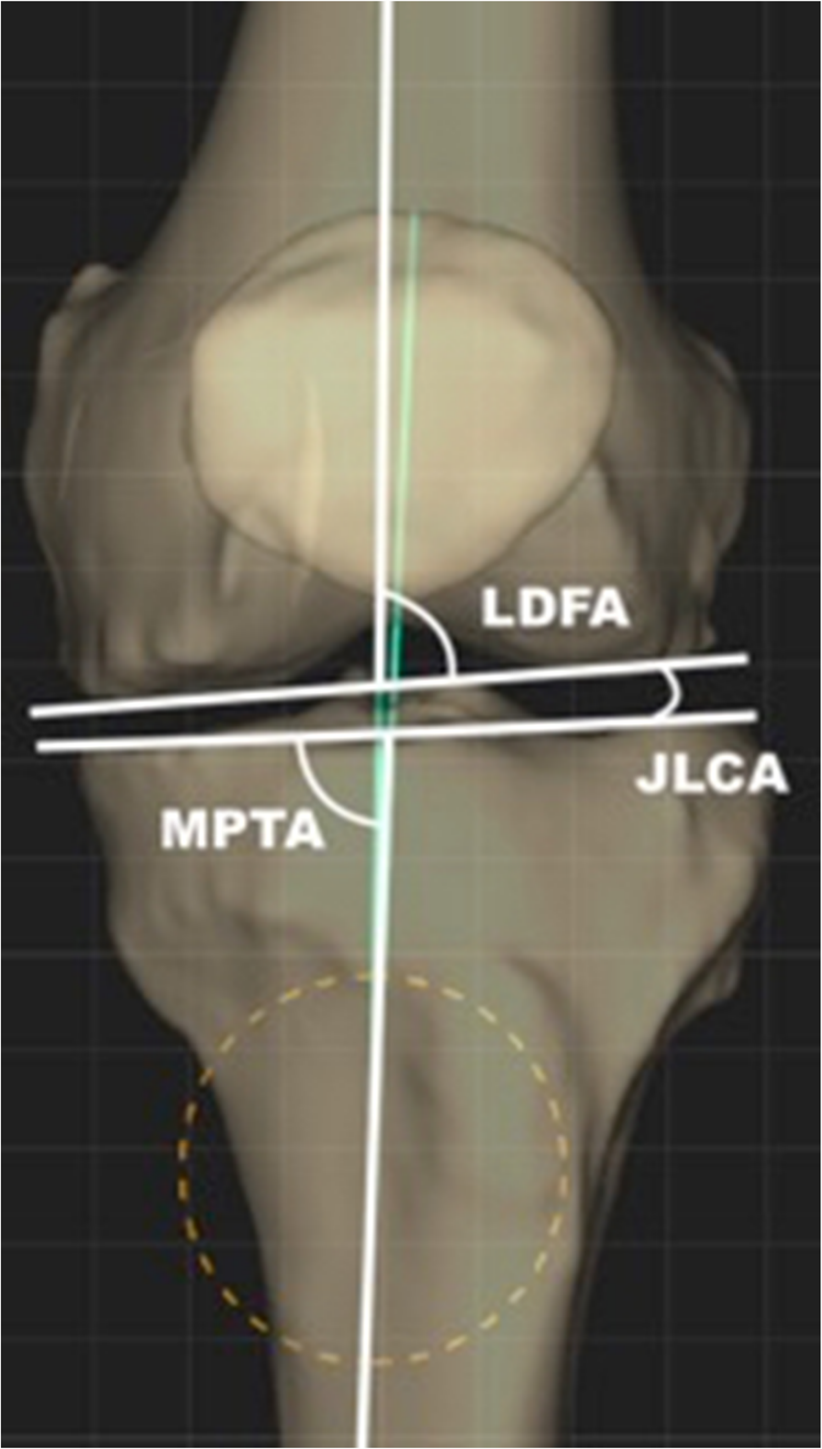
Illustrations of the anatomical measurements. The lateral distal femoral angle (LDFA) was defined as the lateral angle between the femoral axis and the tangent to the femoral condyles. The medial proximal tibial angle (MPTA) was defined as the medial angle between the tibial anatomic axis and the joint line of the proximal tibia. The joint line convergence angle (JLCA) was defined as the angle between the tangent to the subchondral plates of the femoral condyle and the tibial plateau

Then, the hip-knee-ankle (HKA) angle results from the sum of tibial, femoral and intra-articular morphologies, as represented by the medial proximal tibial angle (MPTA), the lateral distal femoral angle (LDFA) and the JLCA [36] (Fig. 1). A positive JLCA value commonly means a varus intra-articular deformity with a medial apex of the JLCA but this standards varies according to studies [4, 14, 44].

Bellemans et al. showed a mean JLCA of 0.51 ± 1.05° with full-leg standing radiographs in a healthy population [5] and a recent review showed that JLCA ranged from 0.47 ± 0.98° for males and 1.9 ± 1.4° for females [33]. In a CT-scan analysis, the non-weight bearing JLCA mean value was 1.09 ± 0.9° [31], which are close to those demonstrated by Chao et al. who reported a normal JLCA ranging from 0° to 2° [8]. In OA knees, Jabalameli et al. demonstrated a mean JLCA of 6.4 ± 3.8° [16]. Indeed, Heijens et al. showed that the postoperative change in the JLCA tended to be larger depending on the initial severity and the advances of OA stages [15] with a positive correlation found between JLCA and preoperative Ahlbäck grade of OA suggesting that the JLCA may be considered as abnormal when the knee joint line converged in more than 3° [39].

Dugdale et al. described an increasing preoperative varus angulation, thought to be due to lateral soft tissue laxity [11] wich corresponds in fact to the double varus due to a separation of the lateral tibio-femoral compartment [34]. Therefore, with advances of the OA stage, a considerably larger varus occur which could not solely be attributed to the bone deformity but also to larger JLCA or intra-articular deformity [9].

Thus, JLCA can be considered to represent the complex interlinking of both surrounding soft tissue laxity and thickness of the remaining cartilage and it can also be influenced by the amount of ligament stretching during weight-bearing, thus the JLCA changes between standing and supine positions [40, 44, 50] and slackness of the lateral soft tissue aggravates varus deformity. Wang et al. also demonstrated that HKA was more valgus after open-wedge high tibial osteotomy (OWHTO) in double-leg position than in single-leg positions and that it would also influence the change of joint line convergence angle [50]. A previous study showed larger JLCA with single-leg weight-bearing position measurements compared to double-leg wei weightbearing [51]. As part of the preoperative alignment assessment, some authors recommend to perform stress radiographs [27, 35, 38, 45, 48] because varus/valgus laxity appears to be important to predict influence of soft tissue laxity on alignment correction [35, 38]. Lee et al. proposed the concept of latent medial laxity by subtracting the JLCA on the weight-bearing standing radiograph from the JLCA on the valgus stress radiograph stress, this is thought to represent the ability of the soft tissue to stretch from a standing position to the valgus stress position [27]. To quantify the instability of the knee joint, other surgeons assessed the change in the JLCA (ΔJLCA) by the difference in JLCA between varus stress and valgus stress radiographs [45, 48]. However, ΔJLCA designation must be interpreted with caution as it varies in its interpretation in each study. The value of ΔJLCA can express a difference between preoperative and postoperative values [13, 23, 28, 35] or a difference between valgus and varus stress radiographs [45, 48] or between supine and standing positions [44]. Mean values of ΔJLCA are indicated on Table 1.

**Table 1:** Summary of reported evaluation methods for JLCA

Author	Year	JLCA in supine position (Mean ± SD)	JLCA in standing position with weight-bearing conditions (Mean ± SD)	JLCA under varus / Valgus stress (Mean ± SD)	ΔJLCA
Kubota et al.	2020	–	3.4 ± 2.2° (single leg)	–	–
Kumagai et al.	2020	2.4 ± 1.6°	5.1 ± 2.0°	–	− 1.9 ± 1.4° (post day 0 – day 2) 0.2 ± 1.2° (1 month – 12 months)
Akasaki et al.	2019	2.1 ± 1.5°	3.8 ± 2.0°	–	1.1 ± 1.0° (pre – post on supine) − 0.7 ± 1.0° (pre – post on standing)
Goshima et al.	2019	–	3.2 ± 1.7°	–	–
Tsuji et al.	2019	–	4.1 ± 2.3° (single leg)	6.0 ± 2.4° / 1.3 ± 2.1°	7.4 ± 2.7° (pre varus – valgus)
Lee et al.	2019	–	3.4 ± 2.2° (double leg)	Latent medial laxity: JLCA in valgus stress – JLCA in weightbearing standing	–
Takagawa et al.	2019	–	4.4 ± 2.3° (single leg)	6.9 ± 2.2° / 1.3 ± 2.5°	8.1° ± 2.8° (pre varus – valgus)
So et al.	2019	2.3°	4.2° (double leg)	6.7° / 0.6 °	1.8° (supine – standing)
Park et al.	2019	–	3.8 ± 1.9°	5.4 ± 2.1°/1.7 ± 1.4°	1.2 ± 1.5° (pre – post)
Kim et al.	2017	–	3.1 ± 1.8°	–	–
Ogawa et al.	2016	–	4.6 ± 2.2° (double legs)	5.6 ± 2.4° / 1.5 ± 1.8°	2.0 ± 1.5° (pre – post)
Lee et al.	2015	–	3.4 ± 2.3°	–	–

*ΔJLCA* JLCA changes between two conditions, *Pre* preoperative, *Post* postoperative

The soft tissue imbalance in patients with varus OA deformity may be influenced by the medial collateral ligament (MCL) and the opening of the medial structure after HTO, the lateral collateral ligament (LCL) is usually relaxed and MCL tensed in medial compartment osteoarthritis with varus deformity. Thus, it has been show that tension of the medial soft tissue was increased without release of MCL after OWHTO [1] but that valgus laxity was increased by release of the MCL during OWHTO and decreased after plate fixation of the osteotomy [41, 43]. In case of valgus deformity correction, the tissue laxity must also be considered and some authors proposed to add MCL reefingplasty to medial CWHTO due to consequent postoperative valgus laxity in 30° and 70° of flexion [30]. Finally, for Park et al. JLCA provided information about cartilage pressure which seems to be unchanged after closing wedge high tibial osteotomy (CWHTO) while the joint space width, provides information about cartilage healing and is slightly increased at 3 months postoperatively [37].

### How does JLCA influence pre-operative planning

**Fig. 2:**
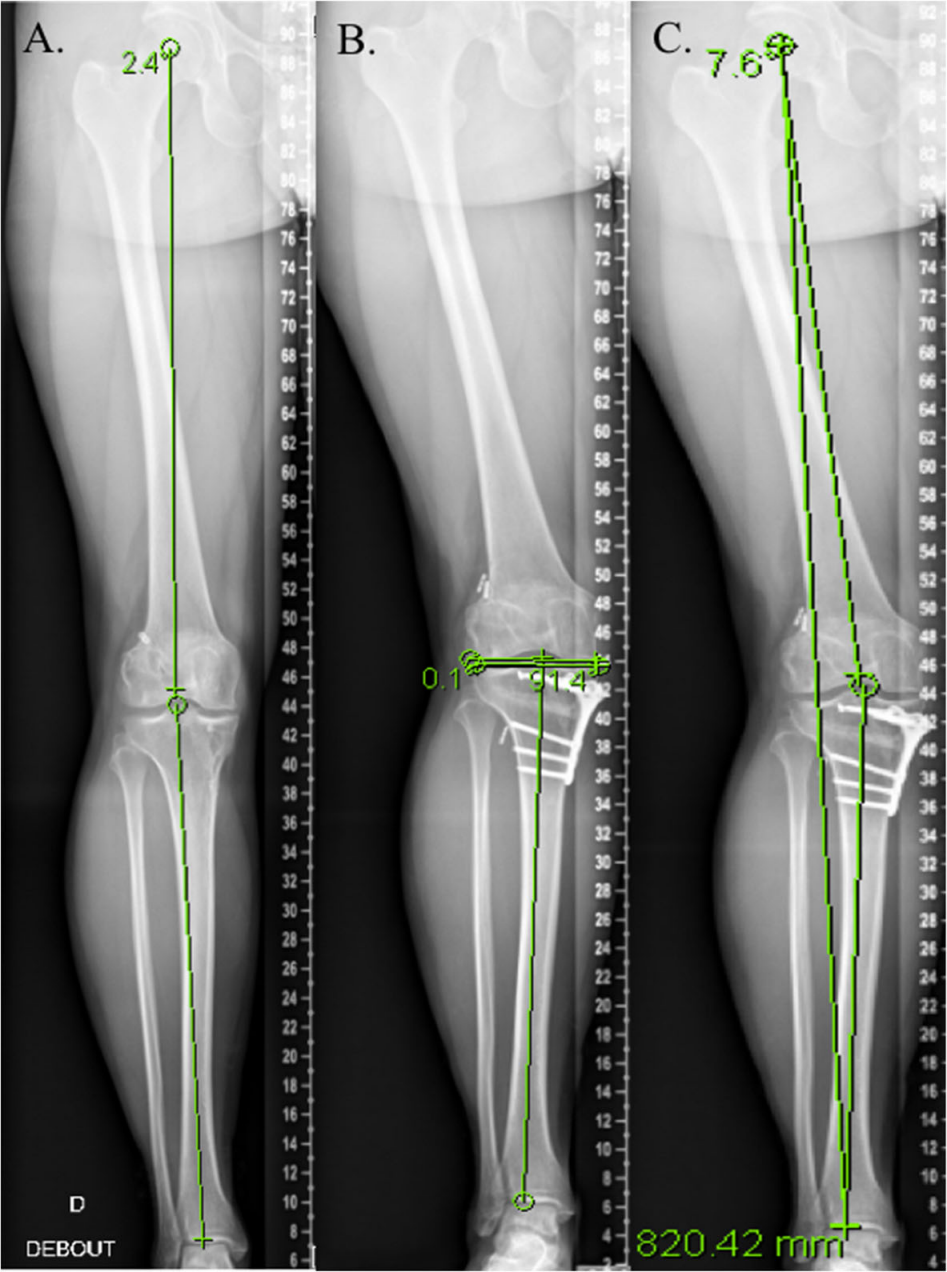
Overcorrection after open-wedge high tibial osteotomy performed without considering the JLCA. **a**. Patient complain medial pain due to previous history of meniscectomy, the preoperative global alignment is 3° varus, Medial proximal tibial angle (MPTA) = 86° and Joint line convergence angle (JLCA) = 5°, **b**. an intended correction of 6° is performed with a postoperative MPTA = 92° but C. the patient had a postoperative overcorrection of 7° valgus with a JLCA = 0°

Many studies have reported differences in pre-operative digital planning and post-operative achieved corrections due to the influence of soft tissue laxity which hasn’t been accounted before osteotomy [19, 24, 44, 48]. The aim of planning is to obtain a predictable mechanical correction, and by not considering the JLCA in these calculations, there is an increased risk of overcorrection. Once the tibial and/or femoral correction has been performed, the previously elevated JLCA, may be reduced to 0, resulting in an overcorrection (Fig. 2).

For Park et al. overcorrection was highly predicted in cases of both greater lateral and medial laxity. They showed that preoperative JLCA values greater than 4° or 1.5° of valgus stress angle were correlated with a greater risk of overcorrection [38]. Other studies have confirmed similar findings, demonstrating that overcorrected groups had larger preoperative JLCA than the acceptable-correction group after OWHTO [13, 35].

Goshima et al. showed that preoperative JLCA in the overcorrected group was 3.9° and 2.6° in the group where acceptable correction was achieved [13], Kim et al. [20] found similar results and advocated that 1° of valgus overcorrection was related with every 2.5° of preoperative JLCA (Table 2). Lee et al. claimed that soft tissue laxity defined by ΔJLCA was correlated with coronal correction error, thus the ΔJLCA was 1.3 ± 1.6° (− 1.2° to 5.6°) in the acceptable-correction group and 2.3 ± 1.9° (− 0.1° to 8.4°) in the over-correction group [28].

**Table 2:** Summary of JLCA influence on osteotomy correction

Author	Year	Soft tissue correction (Mean ± SD)	What is error Correction?	Identified risks factors for correction errors
Kubota et al.	2020	4.4 ± 2.9	Difference between the correction angle during surgery and the radiological correction angle	- Pre MPTA, - JLCA is not associated with ovecorrection
Kumagai et al.	2020	–	ΔJLCA > 2°	- Post JLCA on the day of surgery
Goshima et al.	2019	–	Patients overcorrected group if MPTA > 95°	- Larger pre JLCA
Tsuji et al.	2019	2.0 ± 1.5°	Navigation correction loss > 1.5°	- Higher standing JLCA
Lee et al.	2019	2.6 ± 2.2°	ΔHKA angle in standing long-bone radiographs – ΔHKA angle in navigation	- JLCA in varus stress - Latent medial laxity
Takagawa et al.	2019	3.2°	ΔHKA angle – ΔMPTA defined as the soft tissue correction	- Pre JLCA - JLCA in valgus stress
So et al.	2019	2.0 ± 2.4°	the difference between the change in MA on standing whole-leg radiograph and the coronal correction by navigation > 3°	- Larger ΔJLCA (supine – standing) - Pre varus deformity - Pre JLCA - JLCA in varus stress
Park et al.	2019	5.8 ± 7.4%	Overcorrection if WBL ratio > 10% of the target WBL	- Pre JLCA - Valgus stress angle - ΔJLCA (pre – post)
Ogawa et al.	2016	3.4 ± 2.5°	ΔHKA angle – ΔMPTA defined as the soft tissue correction	- JLCA under varus stess - ΔJLCA (pre – post)
Lee et al.	2015	–	WBL ratios < 57 and > 67% were classified as under- or over-corrections	- Pre JLCA - ΔJLCA (pre – post)

*Pre* preoperative, *Post* postoperative, *ΔJLCA* JLCA changes between two conditions, *ΔHKA angle* Global correction or postoperative, *HKA angle* preoperative HKA angle, *ΔMPTA* Bony correction or preoperative, *MPTA* postoperative MPTA, *WBL* weight bearing lines

Preoperative Kellgren-Lawrence (KL) grade III and IV with moderate tibiofemoral subluxation (< 10 mm) exhibited a larger preoperative JLCA and more valgus overcorrection than patients with minimal subluxation (< 5 mm) [21]. In another study including KL grade II-IV of OA, when the JLCA changed from a mean preoperative value of 4.2° to a mean postoperative value of 2.7°, only 41% of patients achieved an “optimal” postoperative JLCA (defined by the authors by an anatomical JLCA lesser than 2 °). while 15% of patients had an “unacceptable” postoperative JLCA (defined by the authors by a positive JLCA greater than 5 °) and 44% had an “acceptable” postoperative JLCA (2° < JLCA < 5°). In this study, a preoperative JLCA of less than 4° was not associated with an “unacceptable” postoperative JLCA while 3% of patients were classified as “unacceptable” when 4° < JLCA ≤6° and 12% above 6°, implying that a residual varus is left inside the joint after OWHTO especially in patients with large preoperative JLCA (> 6 °) [17].

Soft tissue correction, which leads to correction error, was also associated with preoperative soft tissue laxity including preoperative valgus JLCA values and instability of the knee joint represented by ΔJLCA (varus JLCA minus valgus JLCA) [45].

The influence of the JLCA on preoperative planning and the expected osteotomy correction is indicated in Table 2.

### What suggestions can be made on how to manage JLCA?

Ji et al. showed that with a preoperative JLCA ≤2°, mean postoperative JLCA was 0.41° while in mean preoperative JLCA > 6°, mean postoperative JLCA was 5.27°. The ΔJLCA was 1.1°in the first group and 2.02°. This results suggest that a residual postoperative JLCA is correlated with preoperative values and that OWHTO had limited capability in correcting intra-articular varus [17].

The JLCA may also be influenced by MPTA correction. Akamatsu et al. found that upper tibia corrected to more than 95° of MPTA had higher JLCA values and worse knee function at 2 years compared to those who had MPTA < 95° [2]. This tibial overcorrection may also lead so shear stress in the medial compartment due to resultant joint-line obliquity, this results showed by Nakayama cannot be applied to all patients because it is based on 3D finite element model analysis and inversely, Goshima et al. showed that slight amount of overcorrected MPTA did not affect the clinical outcomes after OWHTO due to compensatory changes in hip and ankle [13]. Kuriyama et al. analysed various computer models of OWHTO and showed that if it is still unclear of what degree of correction is acceptable, tibial overcorrection in the coronal plane results in excessive lateral contact force and abnormal physiological knee kinematics [25].

In order to account for possible overcorrection, Takagawa et al. proposed to anticipate preoperative soft tissue correction by an equation including JLCA and valgus JLCA, even though this model accounted for only 37.1% of the variation in postoperative soft tissue correction [45]. A simple method was proposed by So et al. who recommended that each degree of ΔJLCA, between the supine and standing positions, should be subtracted from the planned amount of correction angle in order to prevent overcorrection [44].

Some other studies have focused on how to predict the JLCA change, as this factor would appear to be far more important to predict which patients will be overcorrected. Thus, Akasaki et al. described the difficulty in predicting postoperative JLCA on preoperative planning radiographs [4]. Instead, they advocated the use of a lateral wedge insole to simulate an OWHTO and measured the resultant JLCA that has been shown to be accurate in measuring the actual postoperative JLCA. If this study considered patient-specific changes in JLCA, only 20° of wedge inclination was used for radiography and did not account for the bony correction needed to each patient.

According to Lee et al., if the latent medial laxity was increased by 1°, the JLCA change will be increased by 0.6°, and when the tibial correction angle was increased by 1°, the JLCA change was increased by 0.2° [27]. Equations for prediction models are given on Table 3.

**Table 3:** Summary of preoperative equations to predict JLCA change or soft tissue correction

Author	Year	Prediction model	Equation	R^2^
Lee et al.	2019	JLCA change	−1.7 + (0.6 × latent medial laxity) + (0.2 × correction angle)	0.49
Takagawa et al.	2019	postoperative soft tissue correction	0.691 × JLCA −0.411 × valgus JLCA − 0.399	0.37
So et al.	2019	postoperative soft tissue correction	planned amount of correction angle – ΔJLCA (ΔJLCA = supine – standing)	0.47
Kim et al.	2017	predictive additional overcorrection	−1.149 + 0.803 x JLCA + 0.176 x LDFA	0.37

*ΔJLCA* JLCA changes between two conditions, *LDFA* lateral distal femoral angle

In our daily practice we have used a simple equation to account for the influence of soft tissue laxity for preventing an overcorrection. A preoperative JLCA ≤2° can be considered as normal and in this case, no soft tissue correction should be considered. In this case, any intra-articular deformity might be totally or partially corrected during lower limb deformity correction.

For a JLCA greater than this, we estimated the value to subtract from the planned correction to be = (JLCA – 2) / 2, to avoid overcorrection.

For example, if a patient had 6° of varus in the proximal tibia and a JLCA of 6° corresponding to an intra-articular varus deformity of 4° (considering normal JLCA lower than 2°), the femur is neutral giving a global mechanical axis of 12° varus (global HKA 168°), then we would consider an valgus overcorrection of 3° with a 13° of correction osteotomy (6° tibia + 3° overcorrection + 4° intra-articular).

This is first likely to lead to an abnormal MPTA with a high risk of joint line obliquity unless double level osteotomy would be considered but secondly the risk would be to have an overcorrection due to the intra-articular deformity (JLCA = 6°) which could only be partially corrected by the osteotomy. In this case, a correction based towards a “kinematic osteotomy” concept would be preferable and we usually aim to only partially correct the intra-articular deformity. Following our formula to obtaining a value to subtract from the planned correction of 13°, the planned intra-articular correction would be 2°: (JLCA – 2) / 2 = (6–2) / 2.

The adequate correction would be 11° in the tibia instead 13° based on the global mechanical axis or 15° if the surgeon wanted to correct the whole intra-articular varus (JLCA = 6°).

So, the HKA would be in a range from 177° (no JLCA correction) to 183° (complete JLCA correction and the better would probably something in-between those values with a reasonable valgus overcorrection.

JLCA may also be considered during the surgery. Kim et al. proposed it to be much easier to apply an intraoperative valgus stress to the knee joint and correct accordingly under fluoroscopy, aiming for a JLCA of 0°–3°. They showed that this method reduced the number of outliers compared to a technique that corrected alignment using the cable method [19]. In this study, the main objective was to consider the JLCA and medial laxity to reduce outliers between planned and obtained corrections.

**Fig. 3:**
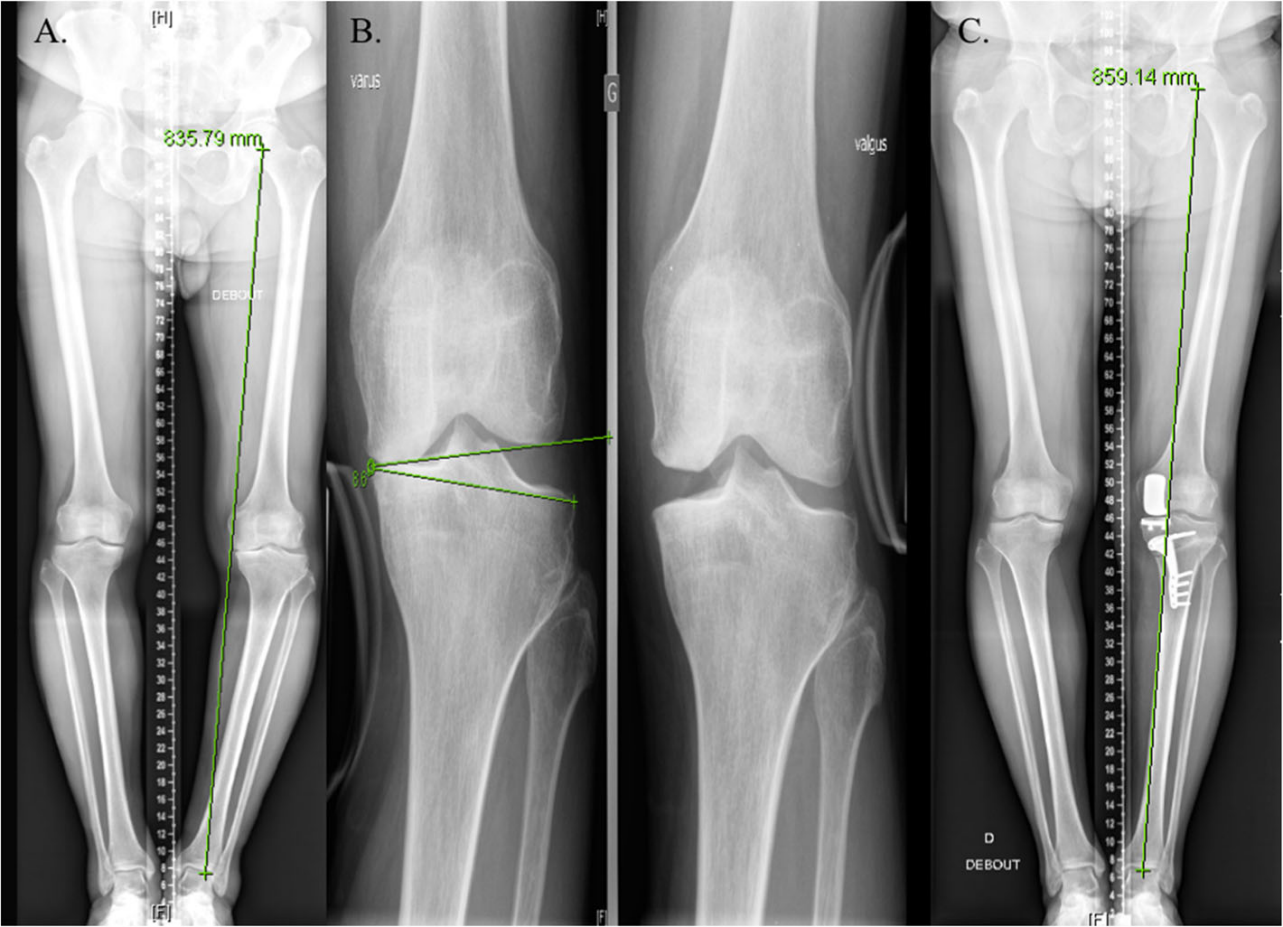
Lower limb varus alignment due to bony and intra-articular deformities. **a**. Patient with a preoperative varus alignment of 14° essentially due to a tibial varus (MTPA = 80°), **b**. The preoperative JLCA measured under varus stress was excessive (JLCA = 17°) suggesting a major postoperative risk of overcorrection **c**. the patient had a an unicompartmental knee prosthesis performed first and simultaneously to his osteotomy allowing to correct the intra-articular and tibial deformities (postoperative MPTA = 86°), the postoperative lower limb morphotype was neutral (HKA angle = 177°)

Chiba et al. have developed the concept of tibial condylar valgus osteotomy (TCVO), arguing that TCVO could alter JLCA in addition to MPTA, making it suitable for patients with a large JLCA but this procedure is technically challenging and has the disadvantage of limited valgus angle correction [9]. Surgeon should also be aware that intra-articular deformity might also require a joint reconstruction procedure (Fig. 3).

Finally, after the surgery, a certain degree of overcorrected MPTA could be acceptable to anticipate secondary correction loss [6, 18] and/or compensatory changes in the hip and ankle joints [7, 13].

## Discussion

The most important findings of the present review are that the preoperative JLCA and ΔJLCA are considered as the most important factors influencing discrepancies between preoperatively planned and postoperative achieved alignments. The required correction angle and method identifying when an overcorrection is anticipated, remains a debate.

Should patients with a larger preoperative JLCA be corrected based only on the mechanical axis even when they risk to have an abnormal postoperative overcorrection or should they better be corrected according to the varus deformity and then reconsider the degree of soft tissue correction?

We propose that the intra-articular deformity defined by the JLCA needs to be considered as well as it changes between weight-bearing or stress conditions. Considering the impact of JLCA on function, lower JLCA was associated with better functional outcomes at early follow-up [17, 48] and Goto et al. demonstrated that large post-operative residual JLCA was independently associated with worsened long-term clinical outcomes (at mean follow-up of 10.2 ± 3.1 years) after CWHTO while a raised medial proximal tibial angle (MPTA) was not a decisive factor that directly affected long-term patient outcomes [14]. These observations reinforce the hypothesis that an advanced osteoarthritis will constitute elements of poor prognosis after osteotomy.

Terauchi et al. observed that a one degree change in the medial proximal tibial angle caused an average of 1.38° of change in the HKA angle showing that a certain soft tissue correction occurred [46]. Other potential contributors wich alters soft tissue imbalance may be related to the general anesthetic because intraoperative assessment after correction is performed using supine fluoroscopy under anesthetized conditions [24], thus, the soft tissue balance around the knee seems to differ between anesthetized and awake conditions.

Options to help guide an accurate outcome include the use of intra-operative navigation systems. Although the correction of the bony anatomy of the proximal tibia is closely correlated with the navigational information, the overall lower limb alignment under weight-bearing situations may be overcorrected if the surgeon depends solely on the navigation system [26].

The intra-articular deformity, represented by the joint space narrowing and the resultant increase in the JLCA significantly increases with the advances of OA [9, 47] but constitutional varus does not affect joint line orientation [49].

Finally, JLCA changes can be anticipated [4, 19, 45] as well as accounting for the changes in soft tissue correction [27, 44]. An equation has been proposed to define the acceptable intra-articular correction with the aim of achieving a more anatomic osteotomy while considering JLCA.

**Fig. 4:**
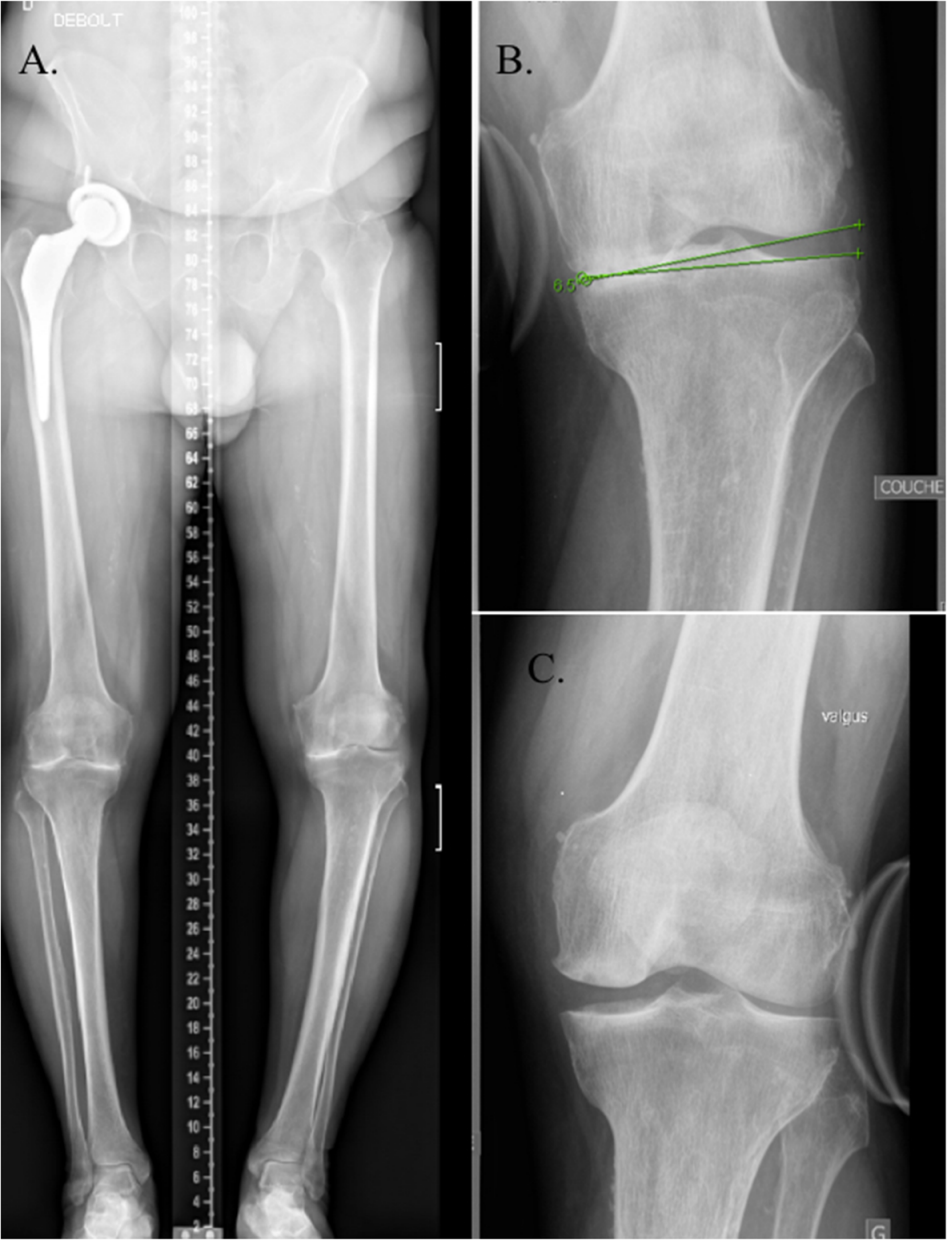
Preoperative X-rays showing soft tissue imbalance. **a**. Patient with a preoperative advanced osteoarthritis and assessment of soft tissue imbalance before osteotomy **b**. Preoperative JLCA under varus stress **c**. Preoperative JLCA under valgus stress

The present review has a considerable number of limitations. Firstly, studies included in this review investigated mostly the alignment on plain long-leg radiographs which are accompanied by the possibility of radiographic measurement errors. Three dimensional CT assessment represents the technique with the highest accuracy in determining anatomical landmarks but would present with the difficulty of assessing coronal alignment and soft tissue laxity in a supine position. Navigation data were used in some cases but this also could lead to mistakes if some of the reference points are not adequately calibrated. In addition, factors were never evaluated in the sagittal or axial plane that might have influence on the correction malalignment. Secondly, many of the studies included a relatively small sample size which can lead to widely varying conclusions. As well as the low sample size, many studies had a short length of follow-up data and it is known that soft tissue and bony corrections possibly changes over a period of time with recurrence of varus as reported by Lee et al. with up to 26% of patients showing recurrence of varus change at 1 year after OWHTO due to significantly larger preoperative JLCA [29]. Thirdly, some studies did not evaluate preoperative X-rays under varus and valgus conditions which could objectify any soft tissue imbalance, particularly in case of advanced osteoarthritis stage (Fig. 4). In the same way, very few studies could have determined how much reduction of correction angle will be required when overcorrection was highly predicted preoperatively which makes clinical relevance and application interpretation really difficult.

Despite the above-mentioned weaknesses, this review is the first to comprehensively show the influence of JLCA on postoperative coronal alignment after osteotomy with particular attention to the risk of unexpected correction errors due to JLCA and soft tissue correction.

## Conclusion

This narrative review provides a detailed overview about the influence and the considerations of joint line convergence angle in view of unexpected correction errors. The JLCA reflect two possible indicators: intra-articular deformity and surrounding soft tissue laxity. The broad spectrum of recent studies showing the influence of JLCA and knee laxity on the postoperative alignment highlights the necessity for a more individualized approach in knee osteotomy. This review also offers the proposals elaborated by surgeons to take account soft tissue correction in the goal of optimal high tibial osteotomy.

## Abbreviations

HKA: Hip–knee–ankle angle
LDFA: Lateral distal femoral angle
MPTA: Medial proximal tibial angle
JLCA: Joint line convergence angle
HTO: High tibial osteotomy
